# Thermal and Hydraulic Performance of CuO/Water Nanofluids: A Review

**DOI:** 10.3390/mi11040416

**Published:** 2020-04-14

**Authors:** Mohammad Yacoub Al Shdaifat, Rozli Zulkifli, Kamaruzzaman Sopian, Abeer Adel Salih

**Affiliations:** 1 Department of Mechanical and Manufacturing Engineering, Universiti Kebangsaan Malaysia, Bangi 43600, Malaysia; shd.mo92@gmail.com (M.Y.A.S.); abeeralnami@yahoo.com (A.A.S.); 2 Solar Energy Research Institute, Universiti Kebangsaan Malaysia, Bangi 43600, Malaysia; ksopian@ukm.edu.my

**Keywords:** CuO/water, nanofluid, thermophysical property, thermal enhancement

## Abstract

This paper discusses the behaviour of different thermophysical properties of CuO water-based nanofluids, including the thermal and hydraulic performance and pumping power. Different experimental and theoretical studies that investigated each property of CuO/water in terms of thermal and fluid mechanics are reviewed. Classical theories cannot describe the thermal conductivity and viscosity. The concentration, material, and size of nanoparticles have important roles in the heat transfer coefficient of CuO/water nanofluids. Thermal conductivity increases with large particle size, whereas viscosity increases with small particle size. The Nusselt number depends on the flow rate and volume fraction of nanoparticles. The causes for these behaviour are discussed. The magnitude of heat transfer rate is influenced by the use of CuO/water nanofluids. The use of CuO/water nanofluids has many issues and challenges that need to be classified through additional studies.

## 1. Introduction

Nanofluids have been used in several industries since their establishment by Choi (1995). Fluids are considered nanofluids when their particle sizes range from 1 to 100 nm [1]. Many researchers have extensively investigated nanofluids in terms of their thermal and dynamic properties, the ability to develop these properties, and the possibilities of using them in industrial applications. Many modelling studies have explored the thermal conductivity of nanofluids because of its strong relationship to heat transfer levels and gained better thermal conductivity using nanofluids than using base fluids [2,3,4,5,6,7,8,9,10,11,12,13,14,15,16,17]. Several experiments have indicated that nanofluids are colloids [18,19,20,21,22] for specific applications, such as various general flow configurations [23,24,25,26,27,28,29] and radiators [30,31,32,33], because of their shear stress, non-Newtonian behaviour, and viscoelastic properties. Many researchers have reported the results of heat transfer for straight tube and microchannel at turbulent nanofluid [16,23,34,35] and laminar [28,36,37] flows. The common base fluids used for nanofluid preparations include engine oil, water and ethylene glycol [38,39,40]. 

Industries have advanced by minimising the size of electronic devices with high processing capacity. However, this approach results in huge challenges in terms of thermal management because of the high thermal densities generated. Nanofluids have become a good alternative for some devices to enhance their thermal management by using them to transport heat and meet their thermal requirements [41]. Nanofluids are also suitable for the aerospace industry. Various industries have explored new technologies, alternative materials and processes to obtain additional thermal enhancements for meeting their tight thermal design requirements [42,43]. In the biomedical industry, nanofluids are modelled as colloidal structures to help antibacterial activities by interacting with complex cellular structures [44,45]. Thermal management is the main factor for the safety and efficiency of fuel engine cars, hybrid and electrical vehicles (EVs). Therefore, many studies have focused on making effective cooling systems using nanofluids for engines and EV batteries [46,47,48].

**Figure 1:**
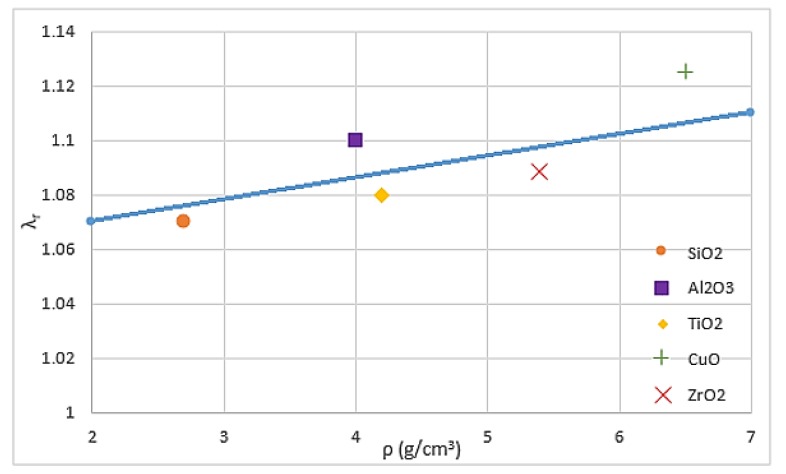
Relative thermal conductivity coefficient versus density of nanoparticles [59].

The thermal and hydraulic properties of nanofluids are the key factors used to provide accurate performance evaluation of their behaviour as working fluid and find methods for enhancing them. Important factors, such as density, viscosity, thermal conductivity and heat transfer coefficient, can be affected by many parameters, including friction factor, Reynolds number, and pump efficiency [49]. Mahbubul et al. [50] and Vajjha and Das [51] investigated the density of various nanofluids and observed that increasing the concentration of nanoparticles and decreasing the temperature led to an increase in density. Mahian et al. [52] reported that high temperature makes the density sensitive to the concentration of nanoparticles. Kulkarni et al. [53] explored the impacts of temperature on SiO_2_, CuO and Al_2_O_3_ water and ethylene glycol-based nanofluids and found that viscosity exponentially increases with the decrease in temperature. Nguyen et al. [54] supported Kulkarni’s result and added that nanoparticle concentration has a significant effect on viscosity. As previously mentioned, most studies have focused on the thermal conductivity of nanofluids because it depends on the concentration, size, shape, and materials of particles [55]. Li et al. [18] and Mintsa et al. [56] indicated that thermal conductivity can be enhanced by decreasing the particle size, whereas Timofeeva et al. [57] showed an opposite point of view. Pryazhnikov et al. [58] stated that no correlation is found between the particle material and the thermal conductivity of nanofluids through systematic measurements. [59,60,61] found a direct relation between the density of nanoparticles and the thermal conductivity of nanofluids through molecular dynamic simulations. This dependence has been confirmed through experiments. Figure 1 shows an approximately linear dependence between the relative thermal conductivity coefficient (*λ*_r_) of water-based nanofluids and the density (*ρ*) of nanoparticles under 100 nm particle size and 2% volume concentration. As shown in the figure, CuO nanoparticles have the highest relative thermal conductivity coefficient due to their highest density, followed by Al_2_O_3_, ZrO_2_, TiO_2_ and SiO_2_, respectively. Al_2_O_3_ nanoparticles takes the second position after CuO nanoparticles although they are not the second highest density nanoparticles, because they have the lowest thermal resistance.

Duangthongsuk and Wongwises [62] conducted an experimental study on TiO_2_ water-based nanofluid using a heat exchanger. They reported that high Reynolds numbers influence the nanofluid temperature, thereby impacting the heat transfer coefficient. By contrast, the heat transfer coefficient is low at high nanofluid temperature. This impact is because of the increase in heat transfer rate with the decrease in nanofluid temperature, thereby increasing the heat transfer coefficient. The base fluid of nanoparticles should be considered because choosing the wrong type of base fluid with nanoparticles probably leads to the deterioration in the heat transfer rate, which can be explained by the mass transfer mechanisms or the increase in viscosity [63,64,65].

Dispersion can be observed in the information regarding the properties and parameters of CuO water-based nanofluids (CuO/water), making them difficult or partially understandable for some researchers. This review paper aims to evaluate some available results for CuO/water nanofluids by clarifying the obtained properties of each respective study. The main objective is to provide the behaviour and trends of CuO/water nanofluids for researchers by showing the attitude of each property in different applications and various conditions. Table 1 shows the studies that used CuO/water nanofluids and their thermal and hydraulic behaviour.

## 2. Thermal Properties

Researchers aim to gain improve thermal performance using CuO/water nanofluids. However, this performance is directly related to many properties, such as heat transfer, thermal conductivity and Nusselt number. These properties enable researchers to evaluate the thermal performance of nanofluids, how they can be increased, and the factors impacting them, which are discussed in the following sections.

### 2.1. Heat Transfer

Scientists and researchers have focused on nanofluids because they serve as a new heat transfer medium to improve the thermal conductivity of fluids by adding small solid particles, leading to the increase in heat transfer for various applications. This section shows the results of most studies that used CuO/water with different concentrations, particle size and models. 

**Figure 2:**
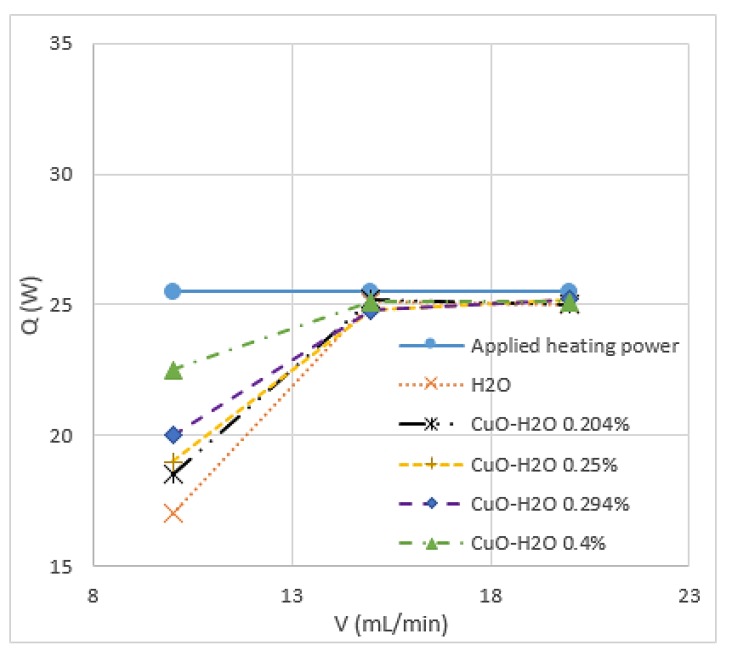
Heat transfer rate (Q) of CuO rectangular nanoparticles with different concentrations in water versus volume flow rate (*V*) [66].

**Figure 3:**
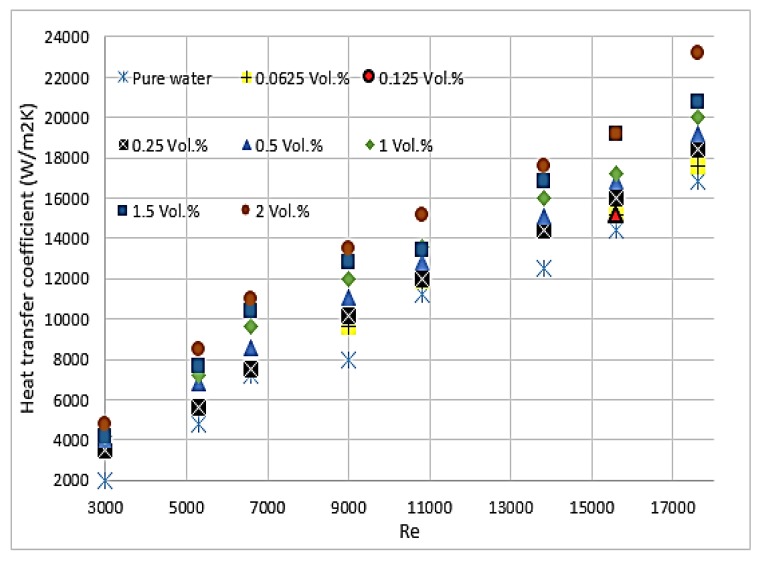
Heat transfer coefficient of CuO spherical nanoparticles with different concentrations (*ϕ*) under various Reynolds numbers [68].

Chein et al. [66] studied CuO/water and indicated that the thermal enhancement, which is gained by adding nanoparticles to the base fluid, depends on particle size, shape, Reynolds number and particle volume fraction. The enhancement of heat transfer using nanofluids was precisely investigated by Heriz et al. [67] through Al_2_O_3_/water (20 nm) and CuO/water (50, 60 nm) with different volume fractions (0.2–3%). They found that heat transfer depends on several factors, including the increase in interactions, fluctuations, chaotic movements of nanoparticles, and thermal conductivity. Jahanpin et al. [37] encountered some limitations for nanofluids using a single-phase model. The obtained results from the single-phase model strongly depend on the adopted thermophysical properties, and the Nusselt number is underestimated in some cases when this model is used compared with models where the properties depend on temperature. Peyghambarzadeh et al. [68] confirmed that increasing the concentration of CuO/water nanoparticles increases the thermal performance of the model. This finding applies to CuO with spherical and rectangular-shaped nanoparticles. As shown in Figure 2, the study of 80 × 20 nm CuO nanoparticles combined with water reported that nanofluids absorb more energy than water and increase with the increase in particle volume fraction [66]. According to [17,69], a small percentage of CuO nanoparticles in water provides a considerable enhancement of the average heat transfer coefficient. Thus, 2% volume fraction of CuO nanoparticles could enhance the heat transfer coefficient up to 57%, as shown in Figure 3. Asirvatham et al. [36] performed an experiment of copper tube under laminar flow to investigate the heat transfer properties of CuO/deionised water. The results agree with the good enhancement of heat transfer coefficient with lower volume concentration of CuO nanoparticles (0.3%) compared with the results of higher concentration (8.2%). They reported that the enhancements are approximately the same, and heat transfer increases with the increase in Reynolds number. [66,68,70] stated that no extra heat absorption is generated with the increase in flow rate. 

**Figure 4:**
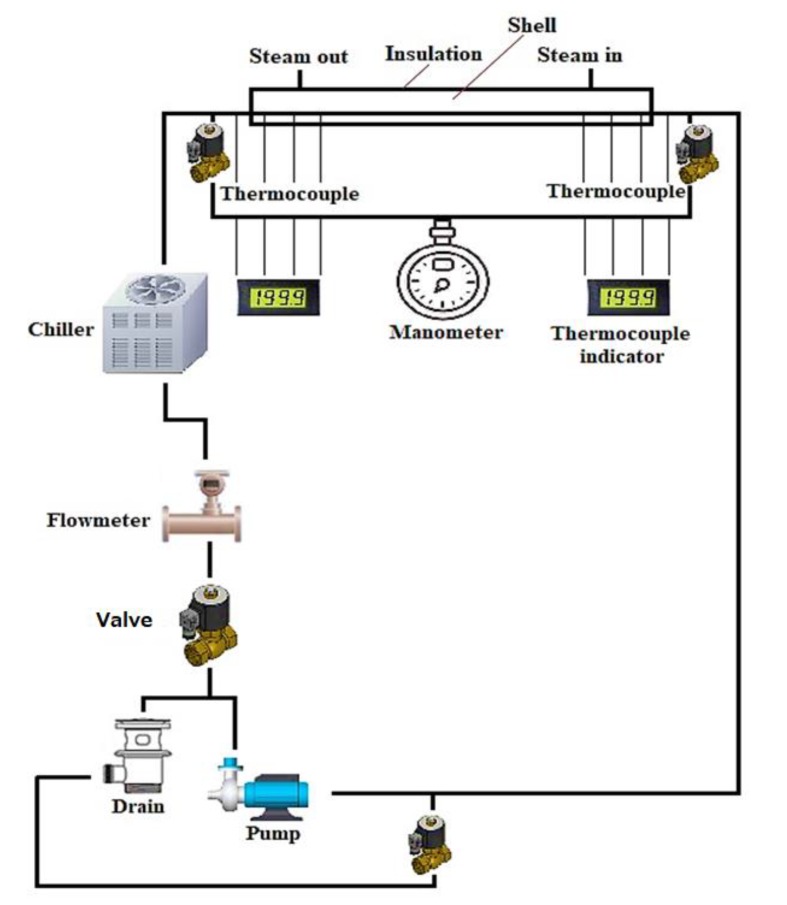
Schematic of experimental circuit.

The type of solid nanoparticles plays an influential role in the percentage of heat transfer enhancements. In [68], Al_2_O_3_/water and CuO/water with different nanoparticle sizes were experimentally investigated using a MCHS to confirm their impacts on heat transfer enhancement. They found that alumina oxide and CuO nanofluids achieve better thermal performance compared with water. Another study [13] used the same nanofluids with rectangular cavity, and proved that Al_2_O_3_/water provides better heat transfer rate than CuO/water. Thus, the highest achieved heat transfer rates are 309 and 298 W for Al_2_O_3_/water and CuO/water, respectively. Heriz et al. [67] created a constant temperature boundary condition by circulating saturated steam in a tube rather than constant heat flux condition, as shown in Figure 4. They indicated that the increase in volume fraction of nanoparticles and Peclet number enhances the heat transfer coefficient. However, the Al_2_O_3_/water nanofluid shows more enhancement in the heat transfer coefficient compared with CuO/water nanofluid. Their findings disagree with the result of Sivakumar et al. [71], who reported that CuO/water nanofluid provides a better heat transfer coefficient compared with Al_2_O_3_/water nanofluid because of the high thermal conductivity of CuO particles. This contradiction could be due to the differences in the used boundary condition, nanoparticle size, and model geometry. 

Surface tension limits the influence of nanofluids to raise the thermal enhancements to some extent. Several studies have effectively reduced surface tension by adding surfactants (hydrophilic and hydrophobic) to the nanofluids. Tran et al. [72] formed a nanofluid environment by suspending carbon nanotubes in distilled water and adding sodium dodecyl sulphate surfactant, resulting in thermal conductivity of 0.75 W/(m·K) for nanotubes with small volume fraction (0.05%), which is 9.36% higher than using only distilled water. However, this high thermal conductivity enhancement does not necessary indicate that the heat transfer has the same enhancement. Byrne et al. [73] used extremely low particle volume fractions (maximum of 0.1%) of CuO/water nanofluid to perform a study on microchannels. They found that adding surfactants to nanofluids only provided a modest heat transfer enhancement compared with pure water, where the highest heat transfer increase is 17% for 0.01% volume fraction.

The movement of nanoparticles inside the nanofluids affects thermal enhancement. The chaotic movement of nanoparticles is considered an important factor to achieve an increase in heat transfer rate [36]. Studying the effectiveness of nanofluids in increasing the heat transfer rate includes a comparison with the performance of other fluids and different alternatives. Zhao et al. [14] developed a 2D numerical model for a square lid-driven enclosure consisting of an intruded rectangular. They showed that the use of CuO/water nanofluid without fins shows a higher heat transfer compared with the utilisation of fins only. Mammari et al. [74] presented different results for the lower thermal enhancement of CuO/water nanofluid compared with fins because of the existence of negative impacts of several factors, such as channel geometry, nanoparticle agglomeration, and insufficient load of nanoparticles.

The heat capacity *C* of a substance, sometimes also called total heat capacity, is the amount of heat required to change its temperature by one Kelvin, and has units of joule per Kelvin (J/K) in the SI system [75,76]. The equation relating thermal energy to heat capacity is Δ*Q = C*Δ*T*, where ΔQ is the thermal energy put into or taken out of the substance, and ΔT is the temperature differential. The heat capacity is therefore an extensive variable and depends simply on the amount of substance. The heat capacity for a mixture of different substances is the sum of the individual heat capacities:

(1) $$C=C_{1}+C_{2}+\cdots$$

The specific heat capacity c of a substance, also named mass-specific heat capacity in science and engineering, is the amount of the heat required to change its temperature of unit mass (one kilogram) of the substance by one Kelvin. The unit of specific heat capacity c in the SI system is the Joule per Kilogram-Kelvin, J/(kg·K). The equation relating heat energy to specific heat capacity is Δ*Q = cm*Δ*T*. The specific heat capacity corresponds to the quotient of heat capacity and mass, or *c = C/m*, where m is the total mass. The specific heat capacity of a mixture of substances is equal to the sum of the individual heat capacities divided by the total mass:

(2) $$c=\frac{C}{m}=\frac{\left(c_{1}m_{1}+c_{2}m_{2}+\cdots \right)}{\left(m_{1}+m_{2}+\cdots \right)}=\omega_{1}c_{1}+\omega_{2}c_{2}+\cdots$$

where *ω_i_ = m_i_/m* is the mass concentration of the *i*th substance.

In the measurement of physical properties, the term “specific” means the measure is an intensive property, wherein the quantity of substance must be specified. For specific heat capacity, mass is the specified quantity (unit quantity). In some books on thermodynamics, the noted specific heat capacity is used for the molar heat capacity. Furthermore, the specific heat capacity is sometimes simply denoted as specific heat. These may cause confusion. In chemistry, the term molar heat capacity c_mol_ of a substance may be used to more explicitly describe the measure of the amount of the heat required to change its temperature of unit quantity of substance (one mole) by one Kelvin. The unit of molar heat capacity c_mol_ in the SI system is the joule per mole-Kelvin, J/(mol·K). The equation relating heat energy to molar heat capacity is Δ*Q = c_mol_n*Δ*T*, where n is the number of moles. The molar heat capacity is related to the heat capacity by *c_mol_ = C/n*, and is related to the specific heat capacity by *c_mol_ = cM*, where M is the molar mass. The molar heat capacity of a mixture of substances is equal to the sum of the individual heat capacities divided by the total number of moles:

(3) $$c=\frac{C}{n}=\frac{\left(c_{mol1}n_{1}+c_{mol2}n_{2}+\cdots \right)}{\left(n_{1}+n_{2}+\cdots \right)}=X_{1}c_{mol1}+X_{2}c_{mol2}+\cdots$$

where *X*_i_ = *n*_i_/*n* is the molar concentration of the ith substance. 

While the “specific heat, *γ*”, of a substance is the ratio of the amount of heat required to raise the temperature of a given mass of the substance through a given range of temperature to the heat required to raise the temperature of an equal mass of water through the same range: *γ = c/c_0_*, where *c*_0_ is the specific heat capacity of water.

The specific volumetric heat capacity, *ρc*, of a substance is the amount of the heat required to change its temperature of unit volume of the substance by one Kelvin, and *ρ* being the density or mass per unit volume. The volumetric heat capacity describes the ability of a given volume of a substance to store internal energy while undergoing a given temperature change, but without undergoing a phase change. The unit of volumetric heat capacity *ρc* in SI system is the Joule per square meter-Kelvin, J/(m^3^·K). The equation relating thermal energy to volumetric heat capacity is Δ*Q = ρcV*Δ*T*, where V is the total volume. The specific heat capacity corresponds to the quotient of heat capacity and volume, or *C/V*. The volumetric heat capacity of a mixture of substances is equal to the sum of the individual heat capacities divided by the total volume:

(4) $$\rho c=\phi_{1}\rho_{1}c_{1}+\phi_{2}\rho_{2}c_{2}+\cdots$$

where *ϕ_i_* *= V_i_/V* is the volume concentration of the ith substance, and *ρ = (ϕ_1_ρ_1_ + ϕ_2_ρ_2_)*, is the density of the mixture. In the case of nanofluid, the specific heat capacity at constant pressure *c*_p_ can be derived from Equation (5), which becomes

(5) $$c_{p,nf}=\frac{\left[\left(1-\phi \right)\rho_{f}c_{p,f}+\phi \rho_{np}c_{p,np}\right]}{\left[\phi_{f}\rho_{f}+\left(1-\phi \right)\rho_{np}\right]}$$

where *ϕ* is the volume concentration of nanoparticle, and the subscripts *nf*, *f*, and np represent for nanofluid, base fluid, and nanoparticle, respectively. The following equation is proposed for determining the specific heat capacity of nanofluid and assessing heat transfer performance of nanofluids [77,78,79]:

(6) $$c_{p,nf}=\left(1-\phi \right)c_{p,f}+\phi c_{p,np}$$

However, it is approximately correct only for dilute suspensions when small density difference exists between base fluid and nanoparticle.

### 2.2. Thermal Conductivity

For the thermal physical properties of nanofluids, thermal conductivity is considered a key property and depends on many factors, such as nanoparticle temperature, volume fraction, size, material, aspect ratio, surfactant and thermal physical properties of base fluid [80]. Many studies have shown that the thermal conductivity of nanofluids is higher than conventional fluids. Several researchers have proposed many theoretical and experimental models to study this type of fluids and find method to improve them.

Researchers have focused on decreasing thermal resistance in the heat transfer of fluids to ensure the high efficiency of thermal systems. Sivakumar et al. [71] proposed a serpentine microchannel of Al_2_O_3_ and CuO spherical nanoparticles (15 nm) with different volume fractions (1–3%, 10%, 20%, 30%) in water for cooling purposes. They concluded that nanofluids can decrease the thermal resistance of MCHS. Another study [81] increased the volume concentration of CuO/water nanofluids to 65% and determined that the thermal conductivity of nanofluids and water are 1.1 and 0.64 W/mK, respectively. This finding indicates that the dispersion of CuO nanoparticles in water enhances the thermal conductivity by two times. Increasing the thermal conductivity and decreasing thermal resistance are related to concentration and flow rate. Thus, the increase in flow rate leads to a reduction in thermal resistance [66]. 

**Figure 5:**
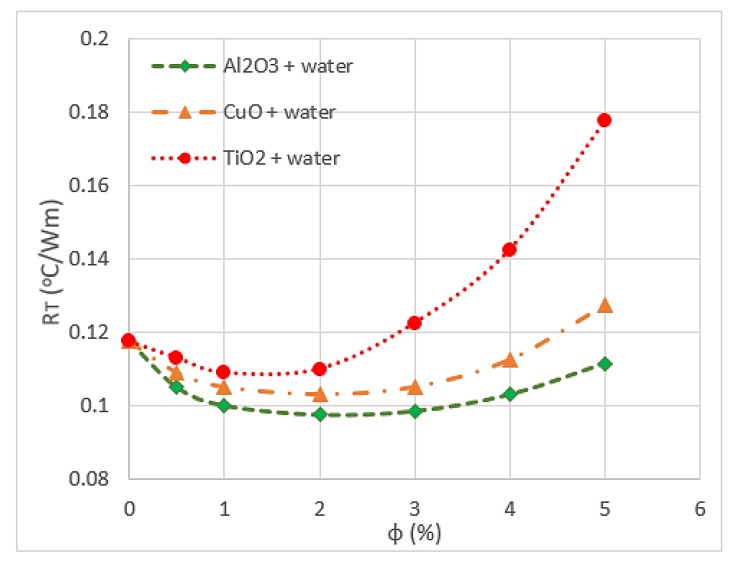
Variation of overall thermal resistance (R_T_) with volume fraction (*ϕ*) [82].

The type of nanoparticle material influences the heat transfer and thermal conductivity of nanofluids. Hung et al. [82] used TiO_2_, Al_2_O_3_ and CuO with water in DL-MCHS to increase the thermal performance of the module. They found that Al_2_O_3_/water nanofluids have the lowest thermal resistance, whereas TiO_2_/water nanofluids have the highest value, followed by CuO/water nanofluids, as shown in Figure 5. This result is because Al_2_O_3_/water nanofluids have low dynamic viscosity and high effective conductivity at different volume fractions. The side effects of increasing the thermal conductivity of nanofluids on other thermophysical properties are the significant drop in heat capacity and increase in viscosity [70]. This observation was obtained by testing CuO/water nanofluids with 4% volume fraction and 30 nm nanoparticle size. Another study showed that changing the volume concentration of CuO/ethylene glycol nanofluids do not affect the heat capacities and remain constant during the test. The authors claimed that no significant impact is observed for particle size because of the large heat capacity of the base fluid [83].

Hamilton and Crosser [84] developed one of the basic models for the prediction of thermal conductivity of nanofluids in which the ratio of conductivity of the solid particles to base fluid is larger than 100, it can be expressed as follows:

(7) $$k_{nf}=\frac{k_{p}+\left(n-1\right)k_{bf}-\phi \left(n-1\right)\left(k_{bf}-k_{p}\right)}{k_{p}+\left(n-1\right)k_{bf}+\phi \left(k_{bf}-k_{p}\right)}k_{bf}$$

where *ϕ* is particle volume fraction, the subscript “*bf*” refers to nanofluid, and “*p*” refers to particle. Koo and Kleinstreuer [6] modified this model as a two–term function for calculating the thermal conductivity of CuO/water nanofluids. The first term is called the static part (Equation (8)) and the second term is due to the Brownian motion which takes into account the effect of particle size, particle volumetric concentration, temperature and properties of base fluid. Therefore, the effective thermal conductivity of a nanofluid can be calculated by Equation (8).

(8) $$k_{nf}=\frac{k_{p}+\left(n-1\right)k_{bf}-\phi \left(n-1\right)\left(k_{bf}-k_{p}\right)}{k_{p}+\left(n-1\right)k_{bf}+\phi \left(k_{bf}-k_{p}\right)}k_{bf}+5\;\times \;10^{4}\beta \phi \rho_{bf}C_{p,bf}\sqrt{\frac{kT}{\rho_{p}d_{p}}ƒ\left(T,\phi \right)}$$

where *n* empirical shape factor, *β* the fraction of the liquid volume which travels with a particle, *d_p_* nanoparticle diameter (*m*), *C_p_* specific heat at constant pressure (J/kg K), *T* temperature (K). The term ƒ(*T*, *ϕ*) in Equation (8) is a function of temperature and particle volume concentration given by Equation (9):

(9) $$ƒ\left(T,\phi \right)=\left(2.8217\;\times \;10^{-2}\phi +3.917\;\times \;10^{-3}\right)\left(\frac{T}{T_{0}}\right)–3.0669\;\times \;10^{-2}-3.91123\;\times \;10^{-3}$$

where “0” is reference temperature. The fraction of the liquid volume which travels with a particle *b* (Equation (8)) is given by Equation (10) for CuO/water nanofluids:

(10) $$\beta =9.881\;\times \;{\left(100\phi \right)}^{-0.9446}$$

In Equations (7) and (8), *n* is empirical shape factor given by *n* = 3/*Ψ* and *Ψ* is the particle sphericity, that is defined as the ratio of the surface area of a sphere with volume equal to that of the particle, to the surface area of the particle. *k*_B_ is Boltzmann constant, *T* is temperature in K, *T*_0_ is reference temperature, and *d_p_* is nanoparticle diameter in m.

### 2.3. Nusselt Number

**Figure 6:**
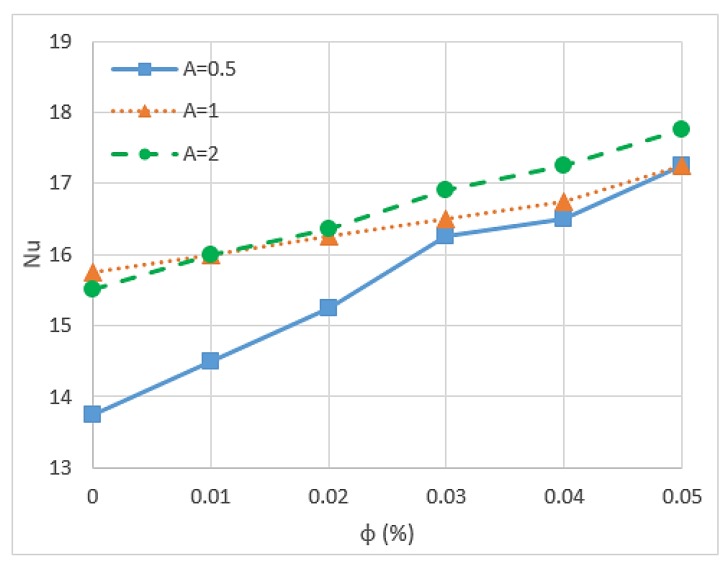
Nusselt number for different aspect ratios and various volume fractions with *Ra* = 10^6^ [86].

**Figure 7:**
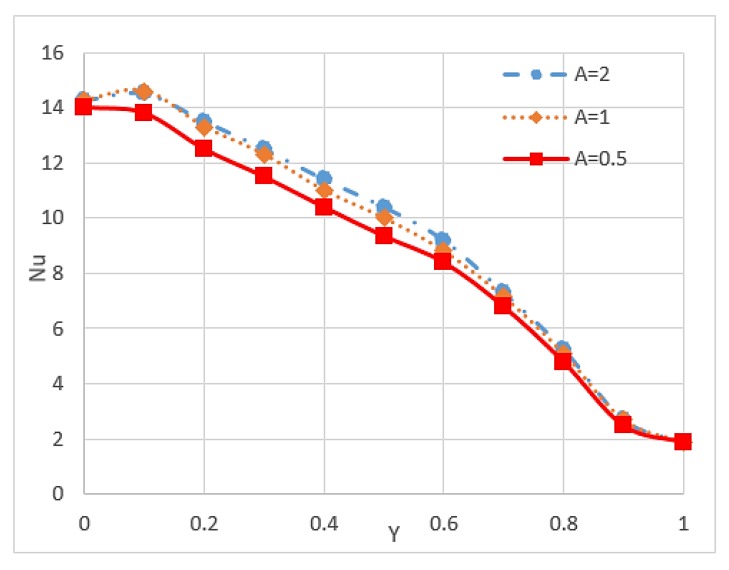
Values of Nusselt number on hot wall (*Y*) at different aspect ratios (*A*) and *Ra* = 10^5^ [86].

Nusselt number is an important parameter because it describes the ratio of convective to conductive heat transfer under the existence of a boundary in a fluid. Conductivity is measured for theoretically motionless fluid, and convection includes diffusion and advection that represent the conduction and fluid motion, respectively. Nusselt number is closely related to the Rayleigh number (Ra) of the fluid [85]. Researchers have found that nanoparticles can increase the Nusselt number of fluid and is affected by some conditions and other factors. Kefayati et al. [86] presented an open rectangular enclosure to study natural convection using Lattice Boltzmann simulation for CuO/water (1–5%) with different aspect ratios. The result indicated that the increment in the volume fraction of nanoparticles and the Ra increase the Nusselt number for the entire range of aspect ratios. However, the increase in aspect ratio leads to a decrease in the Nusselt number, as shown in Figure 6 and Figure 7. The Reynolds number also influences the Nusselt number of nanofluids. Thus, high Reynolds number reduces the Nusselt number of nanofluids because of the possibility of sedimentation and agglomeration of nanoparticles [68]. A study of triangular wavy channel using CuO/water nanofluids under pulsating flow mentioned that the combination of pulsating flow and nanoparticles increase the Nusselt number in the wavy channel compared with the steady flow cases [15].

## 3. Hydraulic Performance

Dispersing CuO nanoparticles into water raises the amount of Reynolds stress, dimensionless mean velocity and velocity fluctuations in all directions and Reynolds number [16]. Thus, the turbulence in the flow field rises with nanoparticles. The temperature follows the turbulence energy and increases at all frequencies. This augmentation is high with high Reynolds number and stream wise direction. The behaviour of CuO/water nanofluids in terms of fluid mechanics are presented in the next sections.

### 3.1. Viscosity and Density

Manufacturers need to predict the behaviour of nanofluids in reality by gathering viscosity data because without correct viscosity can lead to either excessive pumping from the flow path or excessively difficult pumping, thereby resulting in negative impacts on the efficiency of systems and the need of extra costs. The density of nanofluids is important to determine their characteristics. This section focuses on some studies that obtained results about the viscosity and density of CuO/water and their changes under different conditions and factors.

**Figure 8:**
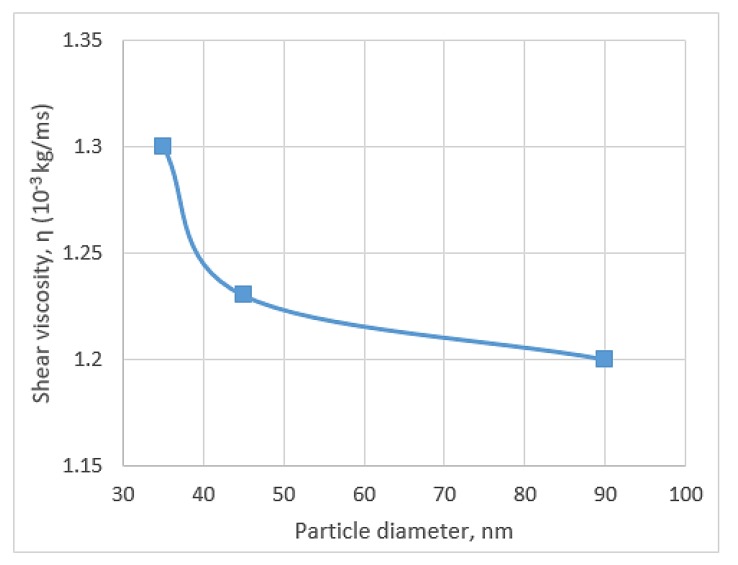
Shear viscosity’s decreasing trend with particle size [87].

Pastoriza-Gallego et al. [87] used different volume concentrations and particle sizes to study the viscosity of CuO/water. They utilised two different samples of synthesised CuO powder 11 ± 3 nm and other 23–37 nm diameter supplied by Nanoarch for 0–10% volume concentrations with 283.15–323.25 K temperature range. They found that the increase in temperature decreases the viscosity at 323.15 K, whereas the viscosity of CuO/water (10%) at 323.15 K becomes approximately equal to that of water at 288.15 K. This result is supported by other researchers, who mentioned that the relative proximity of density and viscosity is the reason for such result [74]. The study of Pastoriza-Gallego et al. obtained many interesting results, such as the strong relation between nanoparticle sizes and viscosity, where small size nanoparticles show large viscosity, and concentration enhances the viscosity for small nanoparticles. However, this tendency cannot be explained for large nanoparticles on the basis of polydispersity and aggregation theories. Figure 8 shows the effects of increasing particle size on the decrease in viscosity. 

The viscosity of suspensions was first studied by Einstein in his classical work. He determined the flow field perturbations caused by the motion of a single particle in a fluid, and obtained the following simple expression for the effective viscosity coefficient:

(11) $$µ={µ}_{0}\left(1+2.5\phi \right)$$

Thus, the viscosity coefficient of a coarse suspension *η* is always greater than the viscosity η_0_ of the base fluid and depends only on the volume concentration of dispersed particles *ϕ*. The interaction between the small concentrations (*ϕ* ≤ 10^−2^) and moderate concentrations (approximately to 10–15%) of particles should be taken into account. For this purpose, Equation (11) has been modified in many studies (see, e.g., [55,88]). The modified formulas can be represented as

(12) $$µ={µ}_{0}\left(1+2.5\phi +k\phi^{2}\right)$$

where the coefficient *k* varies from 4.3 to 7.6.

The viscosity can also be described by the linear relation *η = η_0_(1 + aϕ)*, but, in this case, the coefficient a varies from 4.3 to 22, depending on the type of nanofluid (see, e.g., [88,89,90]); i.e., it is several times the value predicted by Einstein’s theory. In all cases, as the volume (or mass) concentration of nanoparticles increases, a quadratic dependence of the viscosity on *ϕ* is obtained

(13) $$µ={µ}_{0}\left(1+a\phi +b\phi^{2}\right)$$

Several correlations based on different type of nanofluids and base fluids are obtained at different times are given below. One of the first correlations was obtained for a nanofluid with TiO_2_ particles [91]

(13a) $$µ={µ}_{0}\left(1+5.45\phi +108.2\phi^{2}\right)$$

A year later, the following experimental correlation was proposed for a water-based nanofluid containing Al_2_O_3_ nanoparticles [92]

(13b) $$µ={µ}_{0}\left(1+7.3\phi +123\phi^{2}\right)$$

It is worth noting that in the same paper, a different correlation was proposed for a suspension of the same nanoparticles in ethylene glycol

(13c) $$µ={µ}_{0}\left(1+0.19\phi +306\phi^{2}\right)$$

Nanofluid density is calculated from [93]:

(14) $$\rho_{nf}=\frac{m_{nf}}{Vol_{nf}}=\frac{m_{bf}+m_{P}}{Vol_{bf}+Vol_{P}}=\frac{\rho_{bf}Vol_{bf}+\rho_{bf}Vol_{P}}{Vol_{bf}+Vol_{p}}=\left(1-\phi \right)\rho_{bf}+\phi \rho_{P}$$

Indices of “*p*”, “*bf*” and “*nf*” refers to nanoparticles, base fluid, and nanofluid, respectively.

### 3.2. Pressure Drop

**Figure 9:**
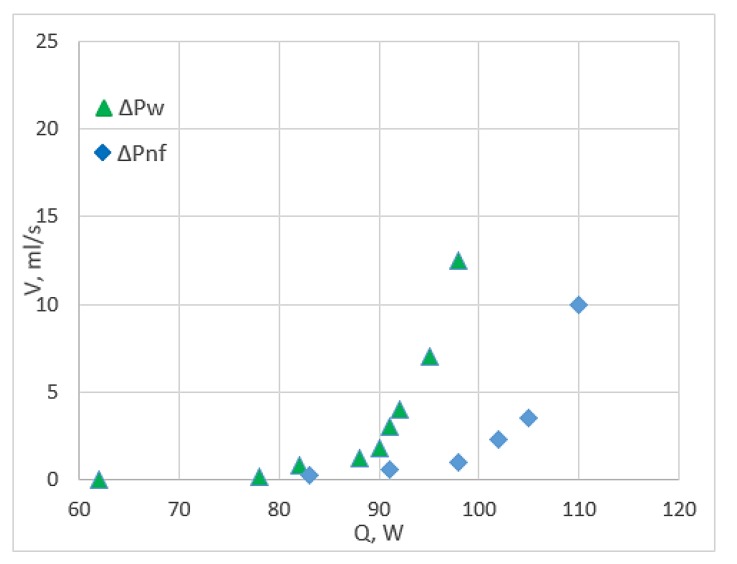
Pressure drop for CuO/water nanofluid and water under different volumes and heat flow rates [70].

Pressure drop is considered a remarkable physical and financial parameter when working on nanofluids. It is the definition of the frictional force, which is made by the resistance to flow and acts on a fluid when it flows through the flow path. CuO/water nanofluids have some degrees of frictional resistance, which is similar to fittings, tubings and valves, resulting in pressure loss. Zarringhalam et al. [17] utilised CuO nanoparticles (40 nm) with various small volume concentrations in a double-tube counter flow heat exchanger. They concluded that pressure drop (∆*P*) is the penalty for using CuO nanoparticles into base liquid, and most experimental data of (∆*P_nf_*/∆*P_w_*) are higher than the standard. This result is supported by [37] and MCHS study [68]. Pantzali et al. [70] reported that the results contradict with the previous results when they studied the pressure drop from the heat load aspect, as shown in Figure 9. The volumetric flow rate of nanofluids is three times lower compared with water for a given specific heat load, leading to a five-fold low pressure drop.

As previously mentioned, many factors increase the pressure drop, and the use of fins with nanofluids becomes effective on the pressure drop of different types of channels. Anwar et al. [94] estimated the lowest pressure drop by utilising CuO/water nanofluids with 100 Pa for 1.5 mm fin spacing heat sink, and the maximum value is 7240 Pa for 0.2 mm fin spacing. The percentage difference of pressure drop ranges from 2.2% to 13.1% for CuO/water nanofluids and water for various fin spacing heat sinks. A study [95] indicated that Reynolds number gradually decreases with the increase in average temperature, and 10% and 50% volume fractions of CuO/water nanofluids decrease the pressure drop.

### 3.3. Sedimentation and Agglomeration

The settling of suspended nanoparticles in the base fluid (sedimentation) and the sticking of nanoparticles to each other or to the surface (agglomeration) are considered in different applications of nanofluids. Nanoparticle sedimentation and the stability of nanofluids play great roles in decreasing thermal conductivity, and this reduction increases with the increase in the diameter and volume concentration of nanoparticles [96]. Focusing on the agglomeration degree of nanoparticles is essential to choose their effective volume concentration [97]. The increase in nanoparticle agglomeration leads to an increase in sedimentation, decrease in thermal performance, and clogging of flowing paths [98].

Each nanofluid has different degrees in deposition on the basis of the mass of solid particles. CuO nanoparticles in water are more prone to deposit than Al_2_O_3_ nanoparticles; this deposition can lead to the deterioration in MCHS because of the high volume concentration of nanoparticles and low flow rates [68]. Researchers have devoted their efforts to reduce the sedimentation and agglomeration of nanofluids, where the use of suspension enhancers (surfactants) in nanofluids provides dispersion and stability of particles, whereas large concentrations encounter significant undesirable foaming [73]. Particle agglomeration can be prevented by making nanofluids at high bulk temperature [66].

## 4. Pumping Power

Pumping power is a sensitive parameter used to determine whether to use or not a specific nanofluid type although that nanofluid has a good thermal performance. Thus, any extra power will lead to a fallen system because each system has limited power consumption. For the heat load aspect, which was considered by Pantzali et al.’s [70] study, CuO/water nanofluids need less volumetric flow rate than water, resulting in low pressure drop and small pumping power. The pumping power rate differs from one nanofluid to another. Hung et al. [82] indicated that Al_2_O_3_/water nanofluids require less pumping power, followed by CuO/water nanofluid and TiO_2_/water nanofluid for 1% nanoparticle volume concentration.

Previous studies have shown that increasing the flow rate increases the convective heat transfer coefficient. However, this option is difficult because it increases the required pumping power, making the system in a good heat transfer condition, whereas deteriorating its power consumption [69]. Adding surfactants remains a good option, as indicated by Byrne et al. [73] that surfactants have no significant pumping power penalty.

## 5. Conclusions

The consequences of using typical nanofluids (i.e., CuO/water) as working fluid for different thermal applications should be studies. All the studies discussed indicate that nanofluids as good candidate of new generation of heat transfer fluids. Investigating the performance of CuO/water enables to understand the factors affecting the thermophysical properties and are deeply dependent on characteristic, temperature and concentration of nanoparticles. The following points show the specific impact of each factor on CuO/water nanofluids:

(1) The heat transfer of nanoparticles depends on their material, and is significantly higher with the existence of fins with CuO/water nanofluids and by increasing many factors, such as flow rate, concentration, Peclet number, and chaotic movement of nanoparticles.
(2) Flow rate, volume fraction, and size of particles strongly influence the thermal conductivity of CuO/water nanofluids.
(3) The increase in Ra increases the Nusselt number, flow rate, concentration, and model geometry.
(4) Viscosity depends on the volume fraction and reduction in particle size. However, increasing the temperature of nanofluids decreases their viscosity.
(5) The existence of fins in the flow path of nanofluids significantly increases pressure drop.
(6) The use of surfactants does not affect the pumping power, increases the temperature of nanofluids, decreases the sedimentation and agglomeration of nanoparticles, and negatively impacts the thermal conductivity of nanofluids.

## Figures and Tables

**Table 1:** Studies that used CuO nanoparticles in base water.

Number	Test Type	Model	Particle Size (nm) ^a^	Volume Fraction (%)	Other Nanofluids Used in the Study	Reference Number
1	Experimental and theoretical	Rectangular cavity	29	40	Al_2_O_3_/water (36 nm, 67%)	13
2	Theoretical	Square lid-driven enclosure	Unknown ^b^	1, 2, 4	-	14
3	Theoretical	Triangular wavy channel	30	5	-	15
4	Theoretical	Rectangular microchannel	40	1.5	-	16
5	Experimental	Double-tube heat exchanger	40	0.0625, 0.125, 0.25, 0.5, 1, 1.5, 2	-	17
6	Theoretical	Triangular microchannel	100	0, 2, 4	-	36
7	Experimental and theoretical	Copper tube	40	0.3	-	37
8	Theoretical	Circular cross-section minichannel	Unknown	10, 30	-	38
9	Experimental and theoretical	Rectangular cavity	80 × 20 rectangular	20–40	-	62
10	Theoretical	Annular copper tube	50, 60	0.2, 1, 2, 2.5, 3	Al_2_O_3_/water (20 nm, similar)	63
11	Experimental and theoretical	Rectangular microchannel heat sink (MCHS)	40	10, 20	Al_2_O_3_/water (20 nm, 10%, 50%)	64
12	Experimental	Circular minichannel	50	0.2	-	65
13	Experimental and theoretical	Miniature plate-fin heat exchanger	30	50	-	66
14	Experimental	Serpentine microchannel	15	1, 2, 3, 10, 20, 30	Al_2_O_3_/water (similar)	67
15	Theoretical	Carbon nanotubes	-	-	-	68
16	Experimental	Plenum microchannel	50	0.5, 1, 10	-	69
17	Experimental	Liquid cold plate	30	10	-	70
18	Experimental	Metallic tube	30	65	-	73
19	Theoretical	Double-layered MCHS (DL-MCHS)	38	0.5, 1, 2, 3, 5	Al_2_O_3_, TiO_2_ water based (similar)	74
20	Theoretical	Open rectangular enclosure	Unknown	1–5	-	76
21	Experimental	Open rectangular enclosure	Supplied by Nanoarch 23–37 Synthesised 11 ± 3	0–10	-	77
22	Experimental	Minichannel heat sink	Unknown	1.5	-	78
23	Experimental	Rectangular microchannel	24	10, 50	-	79

^a^ Particle size: All nanoparticles have spherical shape, whereas others will be written in blank. ^b^ Unknown: Not clearly shown in the original paper of the study.
